# Extracellular traps are evident in Romanowsky‐stained smears of bronchoalveolar lavage from children with non‐cystic fibrosis bronchiectasis

**DOI:** 10.1111/resp.14587

**Published:** 2023-08-30

**Authors:** Amy S. Bleakley, Steven Kho, Michael J. Binks, Susan Pizzutto, Anne B. Chang, Jemima Beissbarth, Gabriela Minigo, Robyn L. Marsh

**Affiliations:** ^1^ Child and Maternal Health Division Menzies School of Health Research, Charles Darwin University Darwin Northern Territory Australia; ^2^ Global and Tropical Health Division Menzies School of Health Research, Charles Darwin University Darwin Northern Territory Australia; ^3^ Research Institute for the Environment and Livelihoods, Faculty of Science and Technology Charles Darwin University Darwin Northern Territory Australia; ^4^ Department of Respiratory and Sleep Medicine Queensland Children's Hospital and Australian Centre for Health Services Innovation, Queensland University of Technology Brisbane Queensland Australia; ^5^ School of Medicine, Faculty of Health Charles Darwin University Darwin Northern Territory Australia; ^6^ School of Health Sciences University of Tasmania Launceston Tasmania Australia

**Keywords:** BAL, bronchiectasis, bronchoalveolar lavage, extracellular traps, light microscopy, paediatric, Romanowsky‐stained

## Abstract

**Background and Objective:**

The importance of extracellular traps (ETs) in chronic respiratory conditions is increasingly recognized but their role in paediatric bronchiectasis is poorly understood. The specialized techniques currently required to study ETs preclude routine clinical use. A simple and cost‐effective ETs detection method is needed to support diagnostic applications. We aimed to determine whether ETs could be detected using light microscopy‐based assessment of Romanowsky‐stained bronchoalveolar lavage (BAL) slides from children with bronchiectasis, and whether the ETs cellular origin could be determined.

**Methods:**

Archived Romanowsky‐stained BAL slides from a cross‐sectional study of children with bronchiectasis were examined for ETs using light microscopy. The cellular origin of individual ETs was determined based on morphology and physical contact with surrounding cell(s).

**Results:**

ETs were observed in 78.7% (70/89) of BAL slides with neutrophil (NETs), macrophage (METs), eosinophil (EETs) and lymphocyte (LETs) ETs observed in 32.6%, 51.7%, 4.5% and 9%, respectively. ETs of indeterminate cellular origin were present in 59.6% of slides. Identifiable and indeterminate ETs were co‐detected in 43.8% of slides.

**Conclusion:**

BAL from children with bronchiectasis commonly contains multiple ET types that are detectable using Romanowsky‐stained slides. While specialist techniques remain necessary to determining the cellular origin of all ETs, screening of Romanowsky‐stained slides presents a cost‐effective method that is well‐suited to diagnostic settings. Our findings support further research to determine whether ETs can be used to define respiratory endotypes and to understand whether ETs‐specific therapies may be required to resolve airway inflammation among children with bronchiectasis.

## Graphical abstract

The burden of paediatric bronchiectasis is an ongoing global challenge. Extracellular traps (ETs) are a potentially important marker for defining paediatric bronchiectasis endotypes. We demonstrate Romanowsky‐stained BAL can be used as a simple and cost‐effective ETs detection method. Using this approach, we determined that ETs were present in BAL slides from 79% of 89 children with non‐cystic fibrosis bronchiectasis. Multiple ET types were observed, with ETs derived from macrophages (METs) and neutrophils (NETs) the most commonly detected types.

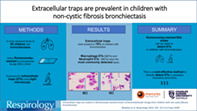

**See related** editorial

## INTRODUCTION

Bronchiectasis is a chronic respiratory disease characterized by abnormal bronchial dilatation and progressive lung function decline.^1^ The burden of bronchiectasis unrelated to cystic fibrosis (henceforth called bronchiectasis) accounts for the greatest number of paediatric bronchiectasis cases globally^2^ and is an important cause of paediatric morbidity among disadvantaged populations with high‐burdens of severe respiratory infections.^3^, ^4^, ^5^

There is increasing interest in defining bronchiectasis endotypes to direct clinical management.^6^ Neutrophil extracellular traps (NETs) are a potentially important endotype marker that could be targeted with existing therapies, including macrolide antibiotics^7^, ^8^, ^9^ and inhaled dornase alfa.^10^ NETs are string‐like structures comprised of extracellular DNA, chromatin and antimicrobial peptides that protrude from neutrophils.^11^, ^12^ NETs can protect against infection by trapping and killing extracellular pathogens; however, they may also promote inflammation and tissue damage if not cleared effectively.^13^, ^14^ In adults with bronchiectasis or chronic obstructive pulmonary disease (COPD), NETs are associated with lung function decline,^15^ disease severity,^8^, ^16^ exacerbation risk^16^ and treatment responses.^8^, ^15^ NETs have been observed in bronchoalveolar lavage (BAL) from children with cystic fibrosis,^10^ protracted bacterial bronchitis (PBB),^17^ bronchiectasis^17^, ^18^ and unspecified causes of chronic cough related to neutrophilic inflammation^10^; however, we are unaware of any studies that have determined NETs prevalence among paediatric populations.

Other immune cells can also release extracellular traps (ETs). Macrophages, the most abundant airway immune cell, can form extracellular traps (METs)^19^, ^20^ and these structures have been detected in BAL from children with cystic fibrosis or other causes of chronic cough.^10^ Eosinophil extracellular traps (EETs) have been observed in respiratory specimens from asthma^21^ and COPD^22^ patients, but their role in disease processes and treatment responses remains poorly understood. There is some evidence that lymphocytes may also produce ETs (LETs)^23^; however, we are unaware of any studies that have reported LETs in respiratory specimens. The presence and clinical significance of METs, EETs and LETs in paediatric bronchiectasis are unknown.

ETs testing is typically done in research laboratories using specialist immunoassays, immunofluorescent staining, confocal microscopy or functional assays.^10^, ^21^, ^22^, ^24^ Such techniques are often unavailable in diagnostic laboratories where BAL testing is limited to differential cell counts performed using Romanowsky‐stains (e.g., Wright or Giemsa stains) and bacterial culture tests. In malaria, Giemsa‐stained slides have been validated for detecting NETs in blood smears.^25^ It is not yet known whether this method could be extended to BAL or other respiratory specimens that may be complicated by mucus, cellular aggregates, biofilms and upper airway secretions.^17^ Availability of a simple ETs detection method that is well‐suited to diagnostic contexts is expected to substantially advance the field by enabling characterization of novel endotype descriptions that are currently absent in paediatric bronchiectasis.

In this study, we aimed to determine: (i) whether ETs could be detected in Romanowsky‐stained BAL slides from children with bronchiectasis; and (ii) whether the ETs' cellular origin could be identified. The secondary aims were to determine ETs prevalence among children with bronchiectasis and to assess whether the presence of ETs in BAL was associated with clinical parameters, BAL inflammatory markers or signs of lower airway infection.

## METHODS

### Study design

A cross‐sectional analysis was performed using archived Romanowsky‐stained BAL slides from 100 children with bronchiectasis or chronic suppurative lung disease (CSLD) who had been prospectively recruited to an observational study of chronic wet cough between August 2010 and October 2013. A sample size of 100 children was arbitrarily chosen a priori, as there were no prior data to inform an ETs prevalence estimate. The date range was selected to include specimens that had previously undergone bacterial culture and cytokine analyses.^26^ A second analysis to assess whether ETs may have been induced as an artefact of sample processing was performed using: (i) peripheral blood from a healthy adult volunteer and (ii) BAL prospectively collected from children recruited to the same observational study between June and August 2021. For all BAL analyses, children were recruited via the paediatric respiratory clinic at Royal Darwin Hospital, Darwin, Australia with written informed consent from their parent/carer, as described previously.^26^

### Diagnostic criteria

Bronchiectasis was diagnosed by a paediatric respiratory physician based on chest high‐resolution computed tomography (cHRCT), as described previously.^1^ CSLD was diagnosed where children had a wet/productive cough for >4 weeks, cHRCT was not done or did not fulfil the criteria of bronchiectasis, and the cough resolved following >2 weeks of antibiotics.^1^ Bronchiectasis severity was scored using a modified‐Bhalla scale, as described previously.^27^

### BAL collection and processing

Bronchoscopy with BAL collection was performed in accordance with the European Respiratory Society guidelines,^28^ as described previously.^29^ Briefly, BAL was collected as two separate lavages and maintained on ice for up to 3 h prior to processing. The first lavage (Lavage‐1) was used for bacterial culture and the second (Lavage‐2) for leukocyte counts and cytokine measures (IL‐6, IL‐8, IL‐1β and IFN‐γ), as detailed in Appendix S1 in the Supporting Information. Slides for differential counts were prepared by applying unfiltered Lavage‐2 containing approximately 5 × 10^4^ leukocytes onto silane‐coated microscope slides (Sigma‐Aldrich, St. Louis, USA) using a cytofunnel™ (ThermoFisher Scientific, Waltham, USA) with centrifugation at 80*g* for 8 min in a Cytospin 4 (ThermoFisher Scientific). Slides were fixed with Quick Dip Fixative (Fronine, Riverstone, Australia) for 3 min, and air dried before staining with Quick Dip stain (Fronine). This stain is a modified‐Romanowsky stain that achieves equivalent leukocyte staining as Giemsa and Wright stains. Leukocyte percentages were calculated from a minimum 300 cells. BAL neutrophilia was defined as a neutrophil percentage >15.

### ETs detection

**FIGURE 1:**
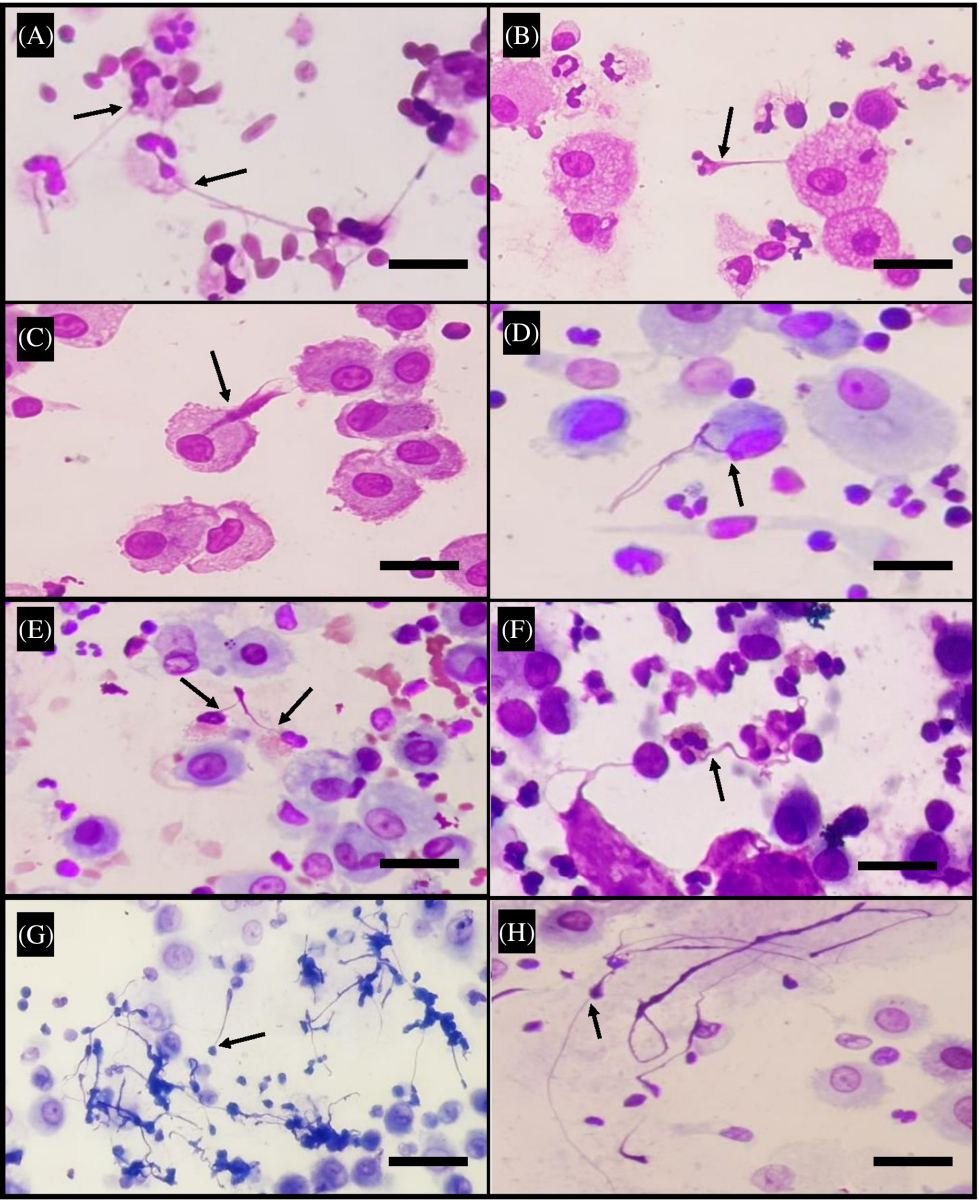
Representative images of extracellular traps (ETs) observed in Romanowsky‐stained bronchoalveolar lavage (BAL) slides from children with bronchiectasis. Representative 400× projection images showing ETs in BAL from children with bronchiectasis. Neutrophil (A,B), macrophage (C,D), eosinophil (E,F) and lymphocyte (G,H) ETs were observed (arrows). Scale bar: 20 μm.

ETs detection was performed according to a previously validated method for Giemsa‐stained peripheral blood smears.^25^ Briefly, the BAL cytoslides were examined by a senior microscopist (S.K.) under high power magnification (400×) on an Olympus CX31 light microscope (Tokyo, Japan). Areas the size of one field of view around the edge of the Cytospin area were excluded. BALs were scored positive for ETs where extracellular string‐like structures with similar coloured staining to the nuclei of surrounding cells (dark blue or purple) were present, with or without contact with nucleated cell(s). Where possible, the cellular origin of each ET was estimated microscopically based on the morphology, size and physical contact with surrounding cell(s), as well as the presence and type of associated granules. ETs observed attached to readily identifiable cells were categorized as either NETs, METs, EETs or LETs (Figure 1). A final category of ‘indeterminate ETs’ was assigned where the cellular origin could not be determined. Where necessary, magnification was increased to 1000× with oil immersion to assess the cellular origin of individual ETs. ETs percentages were calculated from a minimum of 300 leukocytes. To assess ETs quantification across operators, the number of ETs in 15% of slides was reviewed by a second microscopist (A.B.) who was blinded to primary data. The absolute ET count per mL of BAL was estimated by referencing the total leukocyte counts (as previously determined using a haemocytometer).^26^

### Testing for artefactual ETs induction

Additional testing was performed to examine whether ETs were induced during specimen processing. This was done in two stages: (i) using a fresh peripheral blood mononuclear cell (PBMC) suspension to test centrifugation effects; and (ii) using prospectively collected BAL to test temperature and processing time effects. For the PBMC experiment, fresh cells were collected by Ficoll® separation^26^ of peripheral blood from a healthy volunteer. Purified PBMCs were applied to slides using methods identical to those used for BAL. For the BAL experiments, Lavage‐2 was split into two equal aliquots immediately after collection. One aliquot was stored on ice and the other at room temperature during transportation to the laboratory (<15 min). Slides from both aliquots were prepared at time 0 and 3 h after arrival at the laboratory, as described above.

### Statistical analysis

Continuous variables are presented as medians with inter‐quartile ranges (IQR) with groups compared using the Wilcoxon rank sum test. Categorical variables are presented as frequencies and percentages with groups compared using the *χ* ^2^ test. Correlations were determined using Spearman correlation. A *p*‐value <0.05 was considered statistically significant. Concordance was determined using Lin's concordance coefficient and Pearson correlation. Statistical analyses were performed using STATA 16 (Version 15; StataCorp, USA). Hierarchical group average cluster analysis with heat map generation was performed using R (Version 4.0.2; Austria) with the BiocManager,^30^ Heatplus,^31^ gplots,^32^ and RColorBrewer packages.^33^

## RESULTS

### Cohort characteristics

BAL slides from all 100 children were obtained from the archive; however, slides from 11 children were excluded from further analysis due to low sample and/or staining quality. Characteristics of the 89 children included in the analysis are presented in Table 1. Most of the children (84.3%) were aged <4 years (median 2.3 years, IQR 1.6–3.1 years). Eighty‐two children (92.1%) had radiographic evidence of bronchiectasis and 7 (8%) had a clinical diagnosis of CSLD. Given the small number of children with CSLD, data from these children were combined with those of the bronchiectasis group in all subsequent analyses. Bronchiectasis severity ranged up to a modified‐Bhalla score of 20 (median 8, IQR 6–12). BAL neutrophil percentage ranged from 0% to 99.9% (median 10.7%, IQR 4.3%–30.7%), with airway neutrophilia (>15%) evident among 36 (40.4%) children. *Streptococcus pneumoniae*, *Haemophilus influenzae* and *Moraxella catarrhalis* were cultured from 15.3%, 14.1%, 4.7% of BALs, respectively (Table 1).

**TABLE 1:** Clinical characteristics, BAL cytology, culture and extracellular trap data.

Clinical characteristics	
Age, years, median (IQR)	2.3 (1.6–3.1)
Male, *n* (%)	46 (51.7)
Bronchiectasis, *n* (%)	82 (92.1)
Bhalla score, median (IQR)	8 (6–12)
Antibiotics, *n* (%)^a^	46 (51.7)
Azithromycin	33 (37.1)
Other^b^	13 (14.6)
Current cough, *n* (%)	46 (51.7)
Wet	29 (32.6)
Dry	12 (13.5)
Mixed	5 (5.6)

BAL data	
Total leukocyte count × 10^6^/mL, median (IQR)^c^	0.3 (0.2–0.5)
Neutrophil percentage	10.7 (4.3–30.7)
Macrophage percentage	87.0 (61.7–94.3)
Eosinophil percentage	0.7 (0–3.0)
Lymphocyte percentage	0 (0–0.7)
Any pathogen cultured at >10^4^ cfu/mL, *n* (%)^d^	26 (29.2)
*Streptococcus pneumoniae*	13 (15.3)
*Haemophilus influenzae*	12 (14.1)
*Moraxella catarrhalis*	4 (4.7)
*Staphylococcus* spp.	4 (4.7)
*Pseudomonas* spp.	0
Extracellular traps, *n* (%)	70 (78.7)
Macrophage ETs	46 (51.7)
Neutrophil ETs	29 (32.6)
Eosinophil ETs	4 (4.5)
Lymphocyte ETs	8 (9.0)
Indeterminate ETs	53 (59.6)

Abbreviations: BAL, bronchoalveolar lavage; CFU, colony forming units; ETs, extracellular traps; IQR, interquartile range.

^a^ Antibiotic data were available for 87/89 children (two missing data points).

^b^ Other antibiotics included ceftriaxone (*n* = 9), trimethoprim/sulfamethoxazole (*n* = 6), amoxycillin clavulanic acid (*n* = 5), amoxycillin (*n* = 3), flucloxacillin (*n* = 2), benzyl penicillin (*n* = 1), cefotaxime (*n* = 1), metronidazole (*n* = 1) and meropenem (*n* = 1).

^c^ Total leukocyte count data were available for 77/89 children (12 missing data points).

^d^ Culture data were available for 85/89 children (4 missing data points).

### ETs detection from BAL slides

ETs were observed in 70/89 (78.7%) BAL slides (Table 1). Where present, the number of ETs as a percentage of the total leukocyte count was low (median 0.9%, IQR 0.5%–1.5%), equivalent to median absolute ET counts of 4 × 10^3^/mL of BAL (IQR 1–8 × 10^3^/mL). ETs quantification between operators was concordant (Lin's concordance coefficient *r* = 0.94; Pearson *r* = 0.87, *p* = 0.0001; *n* = 13). No ETs were observed in 19/89 (21.3%) BALs.

### Assessing ETs cellular origins

**FIGURE 2:**
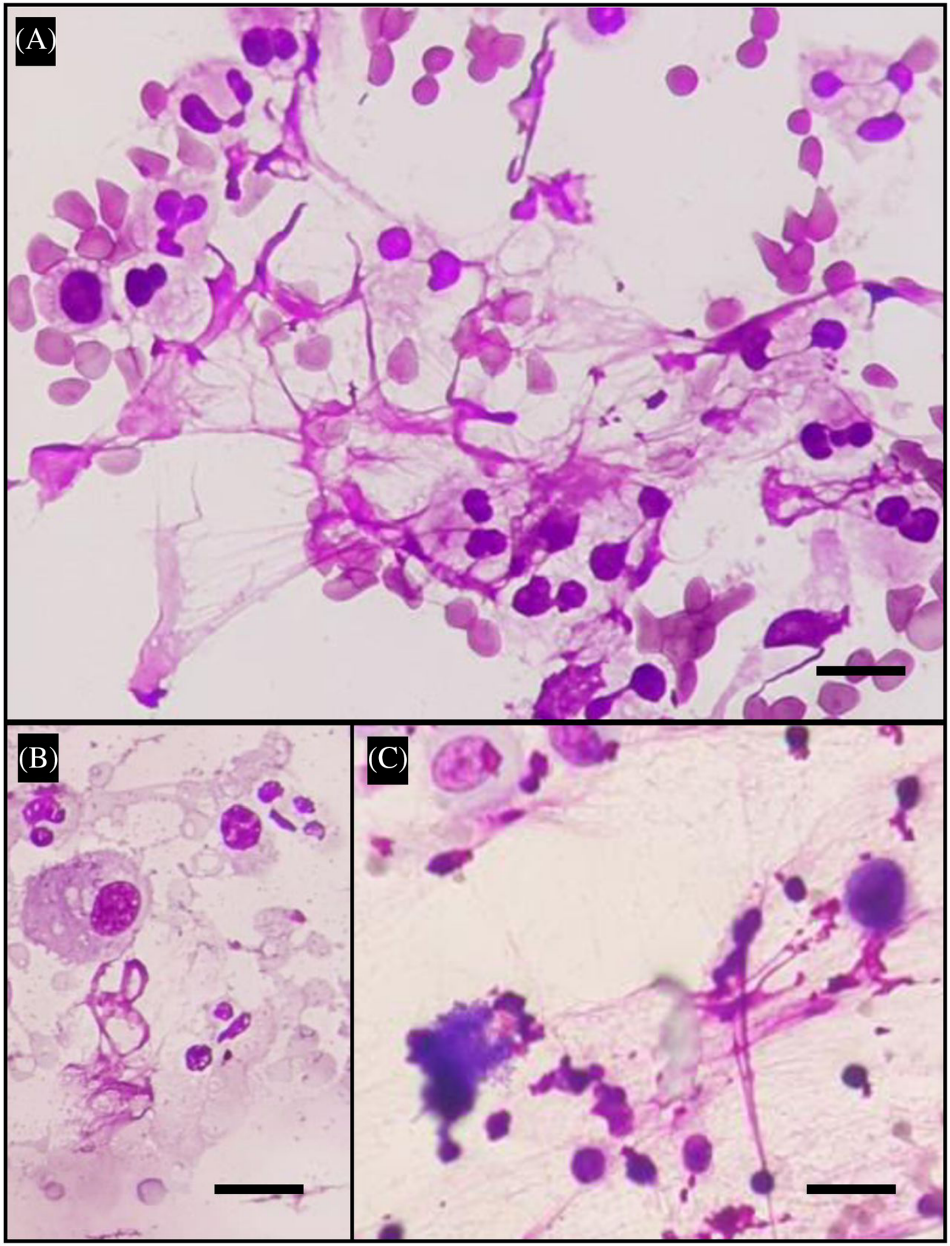
The origin of some extracellular traps (ETs) could not be determined from the Romanowsky‐stained BAL slides (Indeterminate ETs). Representative 400× projection images of Romanowsky‐stained BAL slides showing examples of indeterminate ETs. These ETs were often observed in BAL slides with dense cellularity, resulting in ETs being in contact with multiple potential nucleated cells of origin (A). In some cases, ETs were detached from nucleated cell of origin (B). Unsatisfactory BAL smear, showing clumps of ETs with origin obscured by amorphous debris (C). Scale bar: 20 μm.

A total of 504 ETs were observed across the 89 slides. Of these, the cellular origin (neutrophil, macrophage, eosinophil or lymphocyte) of 65.1% (328/504) could be determined (Figure 1). The remaining 34.9% (176/504) were categorized as indeterminate ETs due to the ET either being: (i) in contact with multiple nucleated cells; (ii) detached from the original cell or (iii) obscured by squamous cells or amorphous debris (Figure 2).

**FIGURE 3:**
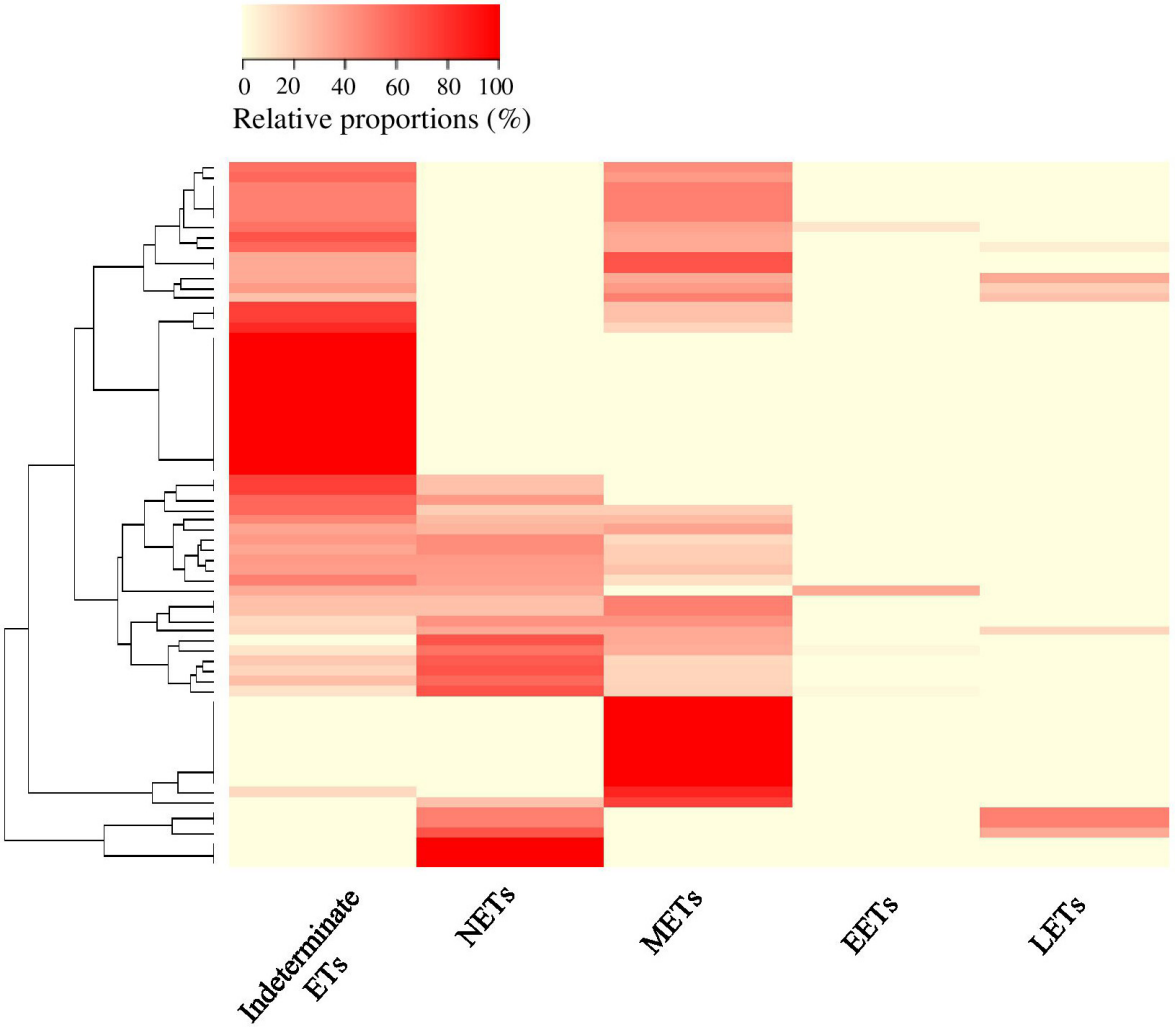
Multiple ET types were frequently co‐detected in BAL. This cluster analysis with heat map shows the relative proportions of ET types (columns) in BAL from each child (rows). The red and beige colours in the heatmap represent relative high and low proportions of ET types, respectively.

Among the 70 ETs‐positive slides, the cellular origin of all ETs in the smear could be determined for 17/70 (24.3%) BALs. In the remaining slides, identifiable and indeterminate ETs were co‐detected in 39/70 (55.7%), whereas only indeterminate ETs were observed in 14/70 (20%; Figure 3).

### ETs originated from multiple lineages

NETs (173/504, 34.3%) and METs (141/504, 28%) were the most common identifiable ETs, observed in 32.6% and 51.7% of BALs, respectively (Table 1, Figure 1A–D). BAL from 13% of children were positive for METs only, and a smaller proportion (3%) only contained NETs (Figure 3). EETs were observed in slides from 4 (4.5%) children (Table 1; Figure 1E,F), all of whom had elevated BAL eosinophil proportions (6.5% [IQR; 4.5%–14%] versus the cohort median 0.7% [IQR; 0%–3%]). In eight (9%) slides, ETs were observed as large clusters that appeared to derive from lymphocytes (LETs; Figure 1G,H). Multiple identifiable ET types were observed in 31.5% (28/89) slides. The most commonly co‐occurring identifiable ETs were METs and NETs, co‐detected in 21.3% (19/89) of BALs (Figure 3). EETs and LETs were always co‐detected with either NETs or METs.

### ETs not induced during processing

Given the high proportion of ETs‐positive BALs and the high number of indeterminate ETs, we next sought to determine whether ET formation may have been triggered during BAL processing. This was done by undertaking quality control experiments using PBMCs from a healthy adult volunteer and prospectively collected BAL specimens from children. ETs were not present in the PBMC cytoslides, indicating that Cytospin processing alone did not induce ETs. In prospective testing of fresh BAL specimens, BAL stored on ice for up to 3 h did not alter the number of ETs observed, consistent with a lack of artefactual ET formation (Table S1 in the Supporting Information).

### Relationships between ETs and clinical markers

We next undertook exploratory analyses to examine whether the presence of ETs was associated with demographic variables (age, bronchiectasis severity, gender and antibiotic use), BAL cytokine markers and culture of respiratory pathogens. Due to the high number of BALs containing indeterminate ETs, these analyses examined relationships between detection of any ETs (including indeterminate ETs) and the clinical variables. There was an association between gender and detection of ETs (*p* = 0.048), with males more likely to have ETs present (40/70, 57%) than females (13/19; 42.9%). No association was observed with age, bronchiectasis severity, antibiotic use or cytokine measures (Table 2). *H. influenzae* was cultured more often from ETs‐positive (11/66, 16.7%) than ETs‐negative (1/19, 5.3%) BAL; however, this difference was not statistically significant (Table 2).

**TABLE 2:** ETs detection was not significantly associated with clinical parameters, BAL inflammatory markers or culture of a respiratory pathogen in univariate tests.

	ETs‐positive	ETs‐negative	*p*‐value
Demographic, median (IQR)	*n* = 70	*n* = 19	
Age (years)	2.3 (1.6–2.9)	1.8 (1.6–5.1)	0.988
Bhalla score (0–20)	8 (5–12)	7 (6–13)	0.447
Demographic, *n* (%)	*n* = 70	*n* = 19	
Male	40 (57.1)	6 (31.6)	**0.048**
Female	30 (42.9)	13 (68.4)	**0.048**
Any antibiotic use^a^	33 (47.8)	13 (72.2)	0.065
BAL cytokine (pg/mL), median (IQR)^b^	*n* = 50	*n* = 12	
IFN‐ɣ	7.8 (3.0–16.7)	15.8 (5.0–42.5)	0.196
IL‐1β	128.5 (46.0–1274.7)	709.2 (36.2–1626.6)	0.858
IL‐6	53.2 (26.1–79.2)	68.2 (33.4–76.3)	0.764
IL‐8	129.4 (33.3–622.4)	47.7 (23.4–268.1)	0.206
BAL culture, *n* (%)^c^	*n* = 66	*n* = 19	
Any bacterial pathogen	19 (28.8)	3 (15.8)	0.254
*S. pneumoniae*	11 (16.7)	2 (10.5)	0.512
*H. influenzae*	11 (16.7)	1 (5.3)	0.208

*Note*: Bold indicates statistically significant differences.

Abbreviations: BAL, bronchoalveolar lavage; ETs, extracellular traps (any type); IFN, interferon; IL, interleukin.

^a^ Antibiotic use data were available for 87/89 children. There was one missing datapoint from ETs‐positive BALs (*n* = 69) and one from ETs‐negative BALs (*n* = 18).

^b^ IL‐8 and IFN‐ɣ data were available for 62/89 children. IL‐1β and IL‐6 were available from 60/89 and 61/89 children, respectively. Missing IL‐1B and IL‐6 datapoints were all from ETs‐negative BALs.

^c^ BAL culture data were available for 85/89 children. Univariate tests were restricted to *S. pneumoniae* and *H. influenzae* only, as too few samples (<5) were positive for other species.

We next examined whether absolute ETs counts (including indeterminate ETs) correlated with BAL cellularity and cytokine concentrations (Table 3). Absolute ET counts were positively correlated with the total leukocyte count (Spearman's *ρ* = 0.526; *p* < 0.0001). This relationship remained apparent when tested against absolute neutrophil (Spearman's *ρ* = 0.438; *p* = 0.0001) and macrophage (Spearman's *ρ* = 0.302; *p* = 0.0076) counts. Absolute ET counts were also positively correlated with IL‐8 (Spearman's *ρ* = 0.485; *p* = 0.0003) and IL‐6 (Spearman's *ρ* = 0.312; *p* = 0.026).

**TABLE 3:** Spearman's correlation between absolute count of any extracellular traps (ET) counts and age, bronchiectasis severity score, BAL cellular characteristics and cytokine levels.

	Absolute ETs × 10^6^/mL		
Demographic	Rho	*p*‐value	*N*
Age (years)	0.145	0.209	77
Bhalla score	−0.062	0.591	77
BAL absolute leukocyte counts (× 10^6^/mL)	Rho	*p*‐value	*N*
Total leukocyte count	0.526	**<0.0001** ***	77
Neutrophils	0.438	**0.0001** **	77
Macrophages	0.302	**0.0076** **	77
Lymphocytes	0.196	0.088	77
BAL cytokines (pg/mL)	Rho	*p*‐value	*N*
IFN‐ɣ (pg/mL)	0.051	0.718	53
IL‐1β (pg/mL)	0.076	0.598	50
IL‐6 (pg/mL)	0.312	**0.026** *	51
IL‐8 (pg/mL)	0.485	**0.0003** **	52

*Note*: Data presented in this table are restricted to the 77/89 children who had BAL total cell count data available. Correlation tests between absolute ET counts and cytokine measures were restricted to a subset of children who had BAL total cell count and cytokine data available. *N* indicates the number of BAL samples included in these tests. Bold indicates statistically significant differences.

Abbreviations: IFN, interferon; IL, interleukin.

*** *p* < 0.0001;

** *p* < 0.01;

* *p* < 0.05.

## DISCUSSION

Use of Romanowsky‐stained BAL to detect ETs presents a simple and cost‐effective method that is well‐suited to diagnostic applications and to informing specialist techniques for confirming ET cellular origins. To our knowledge, this is the first study to demonstrate direct ETs detection from Romanowsky‐stained BAL and the first to examine ETs prevalence among children with bronchiectasis. Among this cohort of 89 children, ETs were detected in 79% of BALs and were derived from multiple leukocyte lineages (predominantly METs and NETs). Absolute ET counts were positively correlated with BAL total cell counts, absolute neutrophil and macrophage counts, IL‐8 and IL‐6.

Our results are consistent with earlier studies describing NETs in sputum and BAL from patients with a range of respiratory conditions,^16^, ^21^, ^34^ including adults with bronchiectasis^8^ and children with cystic fibrosis or other causes of chronic cough.^10^ Results from our experiments with PBMCs and fresh BAL indicate that the high number of ETs‐positive specimens in our study is unlikely to be a specimen processing artefact. This interpretation is supported by the absence of ETs in 21% of the slides examined. Instead, our results support the findings of earlier studies suggesting ETs formation is an important component of the innate response to lower airway infection. Positive correlations between ETs absolute counts and markers of neutrophilic inflammation are consistent with the hypothesis that ETs contribute to airway inflammation among children with bronchiectasis; however, these observations require confirmation with leukocyte‐specific ETs tests.

We did not observe an association between ETs and age, bronchiectasis severity, antibiotic use or culture of bacterial pathogens. We did detect a statistically significant association between ETs and gender; however, replication of this finding is required as a mechanistic explanation of this result is unclear. Further testing is also warranted to confirm the observed absence of an association between ETs detection and culture of respiratory pathogens, especially given higher recovery of *H. influenzae* from ETs‐positive compared to ETs‐negative BALs. Collectively, these findings should be interpreted cautiously as our study was not primarily designed to assess such relationships and few specimens were ETs‐negative.

An important observation from our use of Romanowsky‐stained slides is that multiple leukocyte lineages in BAL can generate ETs, with METs and NETs the most commonly identified types. NETs are increasingly recognized to have clinical importance in chronic airway conditions, including associations with lung function, disease severity, exacerbation risk and treatment responses among adults with COPD or bronchiectasis.^8^, ^15^, ^16^ It is not yet known whether such relationships are generalisable to children with bronchiectasis.

The role of METs in chronic airway diseases is poorly understood; however, in vitro studies have demonstrated MET formation can be triggered by exposure to respiratory pathogens^10^ and neutrophil elastase.^35^ Positive correlations between absolute numbers of both METs and NETs with BAL IL‐8 and IL‐6 suggest a generalized ETosis response related to the underlying drivers of neutrophilic inflammation. Our observation is in accordance with earlier work by King et al.^10^ demonstrating the presence of both NETs and METs in BAL was associated with neutrophilic inflammation among children with cystic fibrosis and other causes of chronic cough. Further studies are needed to determine whether co‐detection of NETs and METs is associated with increased inflammation.

The presence of EETs in BAL was an interesting finding that builds upon earlier reports demonstrating a sub‐population of children with bronchiectasis have lower airway eosinophilia.^29^ EETs have previously been described in asthma,^36^ eosinophilic pneumonia^37^ and chronic rhinosinusitis^38^; however, we are unaware of any studies describing EETs among patients with suppurative lung diseases. The presence of eosinophilic granules aided identification of EETs from Romanowsky‐stained smears; however, follow‐up studies using EETs‐specific testing methods will be necessary to understand the role of these structures in bronchiectasis. It remains to be determined whether the presence of EETs in BAL indicates a distinct bronchiectasis endotype requiring targeted therapy.

The observation of LETs was an unexpected finding. LETs have been considered in acne^39^ and autoimmune diseases^40^ but we are unaware of any reports describing LETs involvement in respiratory conditions. There is some evidence from in vitro studies that LETs may be generated in response to infectious stimuli,^41^ whereas other reports suggest LET‐associated inflammation may disrupt wound healing and promote tissue damage.^39^ Follow‐up testing with lymphocyte‐specific staining methods is needed to confirm the presence of LETs in BAL and sputum and to elucidate the lymphocyte cell types that may be generating these ETs.^40^

Strengths of our study include direct ETs detection using a simple and cost‐effective method that could be used to screen specimens prior to application of specialist leukocyte‐specific methods. Use of Romanowsky‐stained slides is well‐suited to implementation in diagnostic laboratories where Wright and Giemsa‐staining of BAL is performed routinely. Direct ETs detection also enabled the first determination of ETs prevalence among children with bronchiectasis.

Limitations of the study include our use of archived slides. While this supported identification of most ETs, the high proportion of indeterminate ETs restricted analyses of relationships between specific ET types and clinical markers. Specialist staining techniques remain essential to characterize ETs cellular origins and to elucidate relationships between specific ET types and different bronchiectasis endotypes. The study's cross‐sectional design prevented assessment of whether ETs contribute to bronchiectasis disease progression. The study sample size (arbitrarily selected due to a lack of prior data about ETs prevalence) was not powered to assess relationships between specific ET types and the clinical and inflammatory markers. Nevertheless, our results are broadly consistent with earlier reports and provide novel data that informs future research directions. As with other paediatric respiratory studies,^10^ we did not include a healthy control cohort, as BAL was only collected where children underwent bronchoscopy for clinically‐indicated reasons. Our study population also has a high burden of early‐onset CSLD, and therefore it is unclear whether our findings will be generalisable to other paediatric bronchiectasis populations. Further ETs research among paediatric populations with milder suppurative lung diseases, including PBB (an important antecedent of bronchiectasis),^42^ should be conducted to understand the role of ETs across the CSLD disease spectrum. Larger studies are also warranted to determine associations with frequency of exacerbation and lung function and to investigate the impact of antibiotics on ETs production.

In conclusion, multiple ET types are commonly present in BAL from children with bronchiectasis. The presence of ETs can be assessed using Romanowsky‐stained BAL slides, providing a simple and cost‐effective screening method that is well‐suited to implementation in diagnostic laboratories. While this approach facilitates identification of most ETs in BAL, leukocyte‐specific methods remain essential to confirming cellular origins of all ETs. Our results build upon earlier work demonstrating that ETs are a common feature of respiratory conditions characterized by persistent or recurrent lower airway infection. Further research is needed to determine whether different ET types, either alone or in combination, have protective and/or pathogenic roles in paediatric bronchiectasis, and whether ETs‐targeting treatments may be required to resolve persistent airway inflammation.

## AUTHOR CONTRIBUTIONS

**Amy S. Bleakley:** Formal analysis (lead); investigation (equal); writing – original draft (lead); writing – review and editing (equal). **Steven Kho:** Conceptualization (equal); funding acquisition (equal); investigation (equal); methodology (equal); visualization (equal); writing – review and editing (equal). **Michael J. Binks:** Conceptualization (equal); formal analysis (equal); funding acquisition (equal); investigation (equal); supervision (equal); writing – review and editing (equal). **Susan Pizzutto:** Conceptualization (equal); funding acquisition (equal); investigation (equal); methodology (equal); supervision (equal); writing – review and editing (equal). **Anne B. Chang:** Conceptualization (equal); writing – review and editing (equal). **Jemima Beissbarth:** Investigation (equal); project administration (equal); writing – review and editing (equal). **Gabriela Minigo:** Conceptualization (equal); funding acquisition (lead); investigation (equal); supervision (equal); writing – review and editing (equal). **Robyn L. Marsh:** Conceptualization (equal); data curation (equal); funding acquisition (equal); project administration (equal); supervision (equal); writing – original draft (equal); writing – review and editing (equal).

## CONFLICT OF INTEREST STATEMENT

Anne B. Chang reports multiple grants from NHMRC and other fees to the institution from work relating to IDMC membership of an unlicensed vaccine (Glaxo Smith Klein), unlicensed monoclonal antibody for RSV (AstraZeneca) and a COVID‐19 vaccine (Moderna) outside the submitted work. Anne B. Chang also reports fees to the institution from work relating to unlicensed therapies for bronchiectasis (inhaled antibiotics from Zambon and a molecule from BI). The other authors having nothing to disclose.

## HUMAN ETHICS APPROVAL DECLARATION

The study was approved by the Human Research Ethics Committee of the Northern Territory Department of Health and Menzies School of Health Research (HREC# 07/63 and 09/02). Children were recruited via the paediatric respiratory clinic at Royal Darwin Hospital, Darwin, Australia with written informed consent from their parent/carer

## Supporting information


**Appendix S1.** Supporting Information.

**Table S1.** Extracellular traps (ET) observed in prospective bronchoalveolar lavage (BAL) samples from children, processed immediately (time 0 h) and after storage on ice (time 3 h).

**Visual Abstract** Extracellular traps are prevalent in children with non‐cystic fibrosis bronchiectasis

## Data Availability

De‐identified participant data will be made available for respiratory research purposes upon request, subject to appropriate governance agreements including approval from Human Research Ethics Committee of the Northern Territory Department of Health and Menzies School of Health Research.

## References

[1] Chang AB , Bell SC , Torzillo PJ , King PT , Maguire GP , Byrnes CA , et al. Chronic suppurative lung disease and bronchiectasis in children and adults in Australia and New Zealand Thoracic Society of Australia and New Zealand guidelines. Med J Aust. 2015;202(1):21–23. 25588439 10.5694/mja14.00287

[2] McCallum GB , Binks MJ . The epidemiology of chronic suppurative lung disease and bronchiectasis in children and adolescents. Front Pediatr. 2017;5:27. 28265556 10.3389/fped.2017.00027 PMC5316980

[3] Edwards EA , Asher MI , Byrnes CA . Paediatric bronchiectasis in the twenty‐first century: experience of a tertiary children's hospital in New Zealand. J Paediatr Child Health. 2003;39(2):111–117. 12603799 10.1046/j.1440-1754.2003.00101.x

[4] Kapur N , Karadag B . Differences and similarities in non‐cystic fibrosis bronchiectasis between developing and affluent countries. Paediatr Respir Rev. 2011;12(2):91–96. 21458736 10.1016/j.prrv.2010.10.010

[5] Singleton RJ , Valery PC , Morris P , Byrnes CA , Grimwood K , Redding G , et al. Indigenous children from three countries with non‐cystic fibrosis chronic suppurative lung disease/bronchiectasis. Pediatr Pulmonol. 2014;49(2):189–200. 23401398 10.1002/ppul.22763

[6] Flume PA , Chalmers JD , Olivier KN . Advances in bronchiectasis: endotyping, genetics, microbiome, and disease heterogeneity. The Lancet. 2018;392(10150):880–890. 10.1016/S0140-6736(18)31767-7 PMC6173801 30215383

[7] Bystrzycka W , Manda‐Handzlik A , Sieczkowska S , Moskalik A , Demkow U , Ciepiela O . Azithromycin and chloramphenicol diminish neutrophil extracellular traps (NETs) release. Int J Mol Sci. 2017;18(12):2666. 29292737 10.3390/ijms18122666 PMC5751268

[8] Keir HR , Shoemark A , Dicker AJ , Perea L , Pollock J , Giam YH , et al. Neutrophil extracellular traps, disease severity, and antibiotic response in bronchiectasis: an international, observational, multicohort study. Lancet Respir Med. 2021;9(8):873–884. 33609487 10.1016/S2213-2600(20)30504-X

[9] Zhang H , Qiu SL , Tang QY , Zhou X , Zhang JQ , He ZY , et al. Erythromycin suppresses neutrophil extracellular traps in smoking‐related chronic pulmonary inflammation. Cell Death Dis. 2019;10(9):678. 31515489 10.1038/s41419-019-1909-2 PMC6742640

[10] King PT , Dousha L , Clarke N , Schaefer J , Carzino R , Sharma R , et al. Phagocyte extracellular traps in children with neutrophilic airway inflammation. ERJ Open Res. 2021;7(2):00883‐2020. 34164555 10.1183/23120541.00883-2020 PMC8215332

[11] Brinkmann V , Reichard U , Goosmann C , Fauler B , Uhlemann Y , Weiss DS , et al. Neutrophil extracellular traps kill bacteria. Science. 2004;303(5663):1532–1535. 15001782 10.1126/science.1092385

[12] Daniel C , Leppkes M , Muñoz LE , Schley G , Schett G , Herrmann M . Extracellular DNA traps in inflammation, injury and healing. Nat Rev Nephrol. 2019;15(9):559–575. 31213698 10.1038/s41581-019-0163-2

[13] Kaplan MJ , Radic M . Neutrophil extracellular traps: double‐edged swords of innate immunity. J Immunol. 2012;189(6):2689–2695. 22956760 10.4049/jimmunol.1201719 PMC3439169

[14] Twaddell SH , Baines KJ , Grainge C , Gibson PG . The emerging role of neutrophil extracellular traps in respiratory disease. Chest. 2019;156(4):774–782. 31265835 10.1016/j.chest.2019.06.012

[15] Trivedi A , Khan MA , Bade G , Talwar A . Orchestration of neutrophil extracellular traps (Nets), a unique innate immune function during chronic obstructive pulmonary disease (COPD) development. Biomedicine. 2021;9(1):53. 10.3390/biomedicines9010053 PMC7826777 33435568

[16] Dicker AJ , Crichton ML , Pumphrey EG , Cassidy AJ , Suarez‐Cuartin G , Sibila O , et al. Neutrophil extracellular traps are associated with disease severity and microbiota diversity in patients with chronic obstructive pulmonary disease. J Allergy Clin Immunol. 2018;141(1):117–127. 28506850 10.1016/j.jaci.2017.04.022 PMC5751731

[17] Marsh RL , Binks MJ , Smith‐Vaughan HC , Janka M , Clark S , Richmond P , et al. Prevalence and subtyping of biofilms present in bronchoalveolar lavage from children with protracted bacterial bronchitis or non‐cystic fibrosis bronchiectasis: a cross‐sectional study. Lancet Microbe. 2022;3(3):e215–e223. 35544075 10.1016/S2666-5247(21)00300-1

[18] Marsh RL , Thornton RB , Smith‐Vaughan HC , Richmond P , Pizzutto SJ , Chang AB . Detection of biofilm in bronchoalveolar lavage from children with non‐cystic fibrosis bronchiectasis. Pediatr Pulmonol. 2015;50(3):284–292. 24644254 10.1002/ppul.23031

[19] Doster RS , Rogers LM , Gaddy JA , Aronoff DM . Macrophage extracellular traps: a scoping review. J Innate Immun. 2018;10(1):3–13. 28988241 10.1159/000480373 PMC6757166

[20] King PT , Sharma R , O'Sullivan K , Selemidis S , Lim S , Radhakrishna N , et al. Nontypeable *haemophilus influenzae* induces sustained lung oxidative stress and protease expression. PloS One. 2015;10(3):e0120371. 25793977 10.1371/journal.pone.0120371 PMC4368769

[21] Dworski R , Simon HU , Hoskins A , Yousefi S . Eosinophil and neutrophil extracellular DNA traps in human allergic asthmatic airways. J Allergy Clin Immunol. 2011;127(5):1260–1266. 21315435 10.1016/j.jaci.2010.12.1103 PMC3085562

[22] Uribe Echevarría L , Leimgruber C , García González J , Nevado A , Álvarez R , García L , et al. Evidence of eosinophil extracellular trap cell death in COPD: does it represent the trigger that switches on the disease? Int J Chron Obstruct Pulmon Dis. 2017;12:885–896. 28352169 10.2147/COPD.S115969 PMC5359000

[23] Rocha Arrieta YC , Rojas M , Vasquez G , Lopez J . The lymphocytes stimulation induced DNA release, a phenomenon similar to NETosis. Scand J Immunol. 2017;86(4):229–238. 28805301 10.1111/sji.12592

[24] Bendib I , de Chaisemartin L , Granger V , Schlemmer F , Maitre B , Hüe S , et al. Neutrophil extracellular traps are elevated in patients with pneumonia‐related acute respiratory distress syndrome. Anesthesiology. 2019;130(4):581–591. 30676417 10.1097/ALN.0000000000002619

[25] Kho S , Minigo G , Andries B , Leonardo L , Prayoga P , Poespoprodjo JR , et al. Circulating neutrophil extracellular traps and neutrophil activation are increased in proportion to disease severity in human malaria. J Infect Dis. 2019;219(12):1994–2004. 30452670 10.1093/infdis/jiy661 PMC6542661

[26] Pizzutto SJ , Upham JW , Yerkovich ST , Chang AB . High pulmonary levels of IL‐6 and IL‐1β in children with chronic suppurative lung disease are associated with low systemic IFN‐γ production in response to non‐typeable *haemophilus influenzae* . PloS One. 2015;10(6):e0129517. 26066058 10.1371/journal.pone.0129517 PMC4466570

[27] Chang AB , Masel JP , Boyce NC , Wheaton G , Torzillo PJ . Non‐CF bronchiectasis: clinical and HRCT evaluation. Pediatr Pulmonol. 2003;35(6):477–483. 12746947 10.1002/ppul.10289

[28] de Blic J , Midulla F , Barbato A , Clement A , Dab I , Eber E , et al. Bronchoalveolar lavage in children. ERS Task Force on bronchoalveolar lavage in children. European Respiratory Society. Eur Respir J. 2000;15(1):217. 10678650 10.1183/09031936.00.15121700

[29] Pizzutto SJ , Grimwood K , Bauert P , Schutz KL , Yerkovich ST , Upham JW , et al. Bronchoscopy contributes to the clinical management of indigenous children newly diagnosed with bronchiectasis. Pediatr Pulmonol. 2013;48(1):67–73. 22431241 10.1002/ppul.22544

[30] Morgan M . BiocManager: access the bioconductor Project Package Repository. 2021 [accessed 2023 Mar 7] Available from: https://CRAN.R-project.org/package=BiocManager

[31] Ploner A . Heatplus: heatmaps with row and/or column covariates and colored clusters. 2020 [accessed 2023 Mar 7] Available from: https://github.com/alexploner/Heatplus

[32] Warnes G , Bolker B , Bonebakker L , Gentleman R , Huber W , Liaw A , et al. gplots: various R programming tools for plotting data. 2020 [accessed 2023 Mar 7] Available from: https://CRAN.R-project.org/package=gplots

[33] Neuwirth E . RColorBrewer: ColorBrewer Palettes. 2014 [accessed 2023 Mar 7] Available from: https://CRAN.R-project.org/package=RColorBrewer

[34] Dwyer M , Shan Q , D'Ortona S , Maurer R , Mitchell R , Olesen H , et al. Cystic fibrosis sputum DNA has NETosis characteristics and neutrophil extracellular trap release is regulated by macrophage migration‐inhibitory factor. J Innate Immun. 2014;6(6):765–779. 24862346 10.1159/000363242 PMC4201867

[35] Kummarapurugu AB , Zheng S , Ma J , Ghosh S , Hawkridge A , Voynow JA . Neutrophil elastase triggers the release of macrophage extracellular traps: relevance to cystic fibrosis. Am J Respir Cell Mol Biol. 2022;66(1):76–85. 34597246 10.1165/rcmb.2020-0410OC PMC8803356

[36] Lu Y , Huang Y , Li J , Huang J , Zhang L , Feng J , et al. Eosinophil extracellular traps drive asthma progression through neuro‐immune signals. Nat Cell Biol. 2021;23(10):1060–1072. 34616019 10.1038/s41556-021-00762-2

[37] Takeda M , Sakamoto S , Ueki S , Miyabe Y , Fukuchi M , Okuda Y , et al. Eosinophil extracellular traps in a patient with chronic eosinophilic pneumonia. Asia Pac Allergy. 2021;11(3):e24. 34386400 10.5415/apallergy.2021.11.e24 PMC8331252

[38] Hwang CS , Park SC , Cho HJ , Park DJ , Yoon JH , Kim CH . Eosinophil extracellular trap formation is closely associated with disease severity in chronic rhinosinusitis regardless of nasal polyp status. Sci Rep. 2019;9(1):8061. 31147604 10.1038/s41598-019-44627-z PMC6542829

[39] Ouyang K , Oparaugo N , Nelson AM , Agak GW . T cell extracellular traps: tipping the balance between skin health and disease. Front Immunol. 2022;13:900634. 35795664 10.3389/fimmu.2022.900634 PMC9250990

[40] Conceição‐Silva F , Reis CSM , De Luca PM , Leite‐Silva J , Santiago MA , Morrot A , et al. The immune system throws its traps: cells and their extracellular traps in disease and protection. Cell. 2021;10(8):1891. 10.3390/cells10081891 PMC8391883 34440659

[41] Koh CC , Wardini AB , Vieira M , Passos LSA , Martinelli PM , Neves EGA , et al. Human CD8+ T cells release extracellular traps co‐localized with cytotoxic vesicles that are associated with lesion progression and severity in human leishmaniasis. Front Immunol. 2020;11(2663):594581. 33117407 10.3389/fimmu.2020.594581 PMC7578246

[42] Wurzel DF , Marchant JM , Yerkovich ST , Upham JW , Petsky HL , Smith‐Vaughan H , et al. Protracted bacterial bronchitis in children: natural history and risk factors for bronchiectasis. Chest. 2016;150(5):1101–1108. 27400908 10.1016/j.chest.2016.06.030

