# Supplementary material for: Extracellular traps are evident in Romanowsky‐stained smears of bronchoalveolar lavage from children with non‐cystic fibrosis bronchiectasis
Source: Respirology. 2023 Aug 30;28(12):1126–35. doi: 10.1111/resp.14587 (PMC10947271; doi:10.1111/resp.14587)
Supplement: Supplementary file 1 — Appendix S1. Supporting Information. [file RESP-28-1126-s001.docx]

## Microbiology investigations

Semi-quantitative culture identification of *Haemophilus influenzae*, *Streptococcus pneumoniae* and *Moraxella catarrhalis* from BAL were performed at the Menzies School of Health Research Laboratory. Children were considered to have signs of lower airway infection where at least one of these pathogenic bacteria (*Streptococcus pneumoniae, Haemophilus influenzae*, and *Moraxella catarrhalis)* was cultured at >10^4^ colony forming units (CFU)/mL of BAL. *Staphylococcus* spp. and *Pseudomonas* spp*.* were cultured as above but species identification was not performed.

## Quantification of airway cytokines

Airway cytokines were measured in BAL supernatant at the Menzies School of Health Research Laboratory. Firstly, Lavage-2 aliquots were centrifuged at 400 x g for 8 mins and the supernatant stored at -80°C prior to cytokine testing. In-house dissociation-enhanced lanthanide fluorescent immunoassays (DELFIA) were used to assay interleukin (IL)-6, IL-8, IL-1β, and interferon (IFN)-γ levels, as described previously.^1^ The limit of detection for all cytokine assays was 10 pg/ml; samples with concentrations below the limit of detection were assigned the value of 1 pg/ml.

^1^ Pizzutto, S.J., J.W. Upham, S.T. Yerkovich, et al., *High Pulmonary Levels of IL-6 and IL-1β in Children with Chronic Suppurative Lung Disease Are Associated with Low Systemic IFN-γ Production in Response to Non-Typeable Haemophilus influenzae.* PloS one, 2015. **10**(6): p. e0129517-e0129517.
