# Supplementary material for: Extracellular traps are evident in Romanowsky‐stained smears of bronchoalveolar lavage from children with non‐cystic fibrosis bronchiectasis
Source: Respirology. 2023 Aug 30;28(12):1126–35. doi: 10.1111/resp.14587 (PMC10947271; doi:10.1111/resp.14587)
Supplement: Supplementary file 2 — Table S1. Extracellular traps (ET) observed in prospective bronchoalveolar lavage (BAL) samples from children, processed immediately (time 0 h) and after storage on ice (time 3 h). [file RESP-28-1126-s003.docx]

**Table S1**. Extracellular traps (ET) observed in prospective bronchoalveolar lavage (BAL) samples from children, processed immediately (Time 0 hrs) and after storage on ice (Time 3 hrs).

| BAL No | ET count (per 300 WBC) | |
| --- | --- | --- |
|  | Time 0 hrs | Time 3 hrs |
| 1 | 53 | 44 |
| 2 | 7 | 3 |
| 3 | 5 | 8 |
| 4 | 16 | 19 |
| 5 | 34 | 28 |
