# Supplementary material for: Extracellular traps are evident in Romanowsky‐stained smears of bronchoalveolar lavage from children with non‐cystic fibrosis bronchiectasis
Source: Respirology. 2023 Aug 30;28(12):1126–35. doi: 10.1111/resp.14587 (PMC10947271; doi:10.1111/resp.14587)
Supplement: Supplementary file 3 — Visual Abstract Extracellular traps are prevalent in children with non‐cystic fibrosis bronchiectasis [file RESP-28-1126-s002.pdf]

Extracellular traps are prevalent in children with non-cystic fibrosis bronchiectasis

METHODS

A cross-sectional study of **89 children** with **bronchiectasis**

Using **Romanowsky-stained** bronchoalveolar lavage (BAL) slides

Examined for **extracellular traps (ETs)** using **light microscopy**

RESULTS

Extracellular traps were present in **79%** of children with bronchiectasis

Macrophage ETs (METs) and **Neutrophil ETs** (METs) were the **most commonly detected** types.

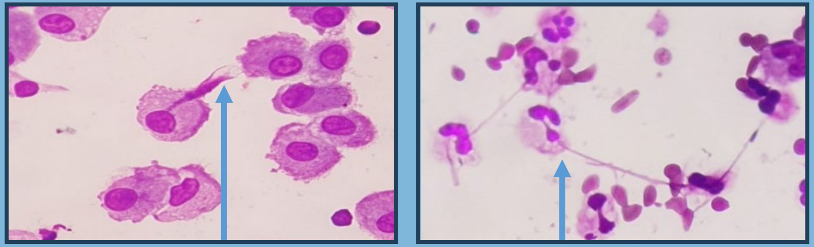

SUMMARY

Romanowsky-stained BAL slides can be used to **detect ETs** in children with bronchiectasis.

This is a **cost-effective method** to directly **detect ETs** in paediatric respiratory specimens.

$$$

menzies school of health research | CHARLES DARWIN UNIVERSITY AUSTRALIA

Respirology

Extracellular traps are evident in Romanowsky-stained smears of bronchoalveolar lavage from children with non-cystic fibrosis bronchiectasis

Bleakley et al. *Respirology* 2023; DOI: 10.1111/resp.14587
